# Walking with wider steps changes foot placement control, increases kinematic variability and does not improve linear stability

**DOI:** 10.1098/rsos.160627

**Published:** 2017-09-13

**Authors:** Jennifer A. Perry, Manoj Srinivasan

**Affiliations:** 1 Department of Mechanical and Aerospace Engineering, The Ohio State University, Columbus, OH 42310, USA; 2 Department of Physical Medicine and Rehabilitation, University of Virginia, Charlottesville, VA 22908, USA

**Keywords:** walking, stability, foot placement, feedback control, step width

## Abstract

Walking humans respond to pulls or pushes on their upper body by changing where they place their foot on the next step. Usually, they place their foot further along the direction of the upper body perturbation. Here, we examine how this foot placement response is affected by the average step width during walking. We performed experiments with humans walking on a treadmill, both normally and at five different prescribed step widths. We prescribed step widths by requiring subjects to step on lines drawn on the treadmill belt. We inferred a linear model between the torso marker state at mid-stance and the next foot position. The coefficients in this linear model (which are analogous to feedback gains for foot placement) changed with increasing step width as follows. The sideways foot placement response to a given sideways torso deviation decreased. The fore–aft foot placement response to a given fore–aft torso deviation also decreased. Coupling between fore–aft foot placement and sideways torso deviations increased. These changes in foot placement feedback gains did not significantly affect walking stability as quantified by Floquet multipliers (which estimate how quickly the system corrects a small perturbation), despite increasing foot placement variance and upper body motion variance (kinematic variability).

## 1. Introduction

Humans modulate their foot placement during active stabilization of walking [1–3]. Foot placement allows humans to change their leg direction, thereby changing their leg force direction during walking [2]. For instance, say a walking person is pushed rightward when their left foot is on the ground. To recover from this perturbation, they may place their right foot further to the right than normal, so that the right leg force has a greater leftward component, acting as a restoring force [2,4]. Such foot placement reactions have been found in response to perturbations to the upper body [3,5] and have been inferred from steady-state walking data [6–8]. Persistent pseudorandom visual and mechanical perturbations also increase step length and step-width variability, suggesting (but not demonstrating) the use of foot placement in response to the perturbations [9–11].

Here, we examine how such foot placement responses change while walking with step widths larger and smaller than one's natural step width [12–14]. Specifically, as in [6], we fit a linear model to the variability in steady-state walking data, inferring a relationship between the pelvis state (or centre of mass state) at mid-stance and the position of the next stance foot. Such a linear model may represent how the foot placement is modulated in response to deviations in centre of mass state. We find that the coefficients in this linear model—the effective ‘foot placement control gains’—change systematically with step width. One might expect that wider steps improve stability by increasing the base of support (at least during double stance), thereby reducing the need for foot placement modulations [15,13]. However, we find that a simple measure of walking linear stability (namely, Floquet multipliers [16,17]) is largely unaffected, but that foot placement and upper body motion standard deviations are increased.

## 2. Methods

### 2.1. Experiments

**Figure 1.**
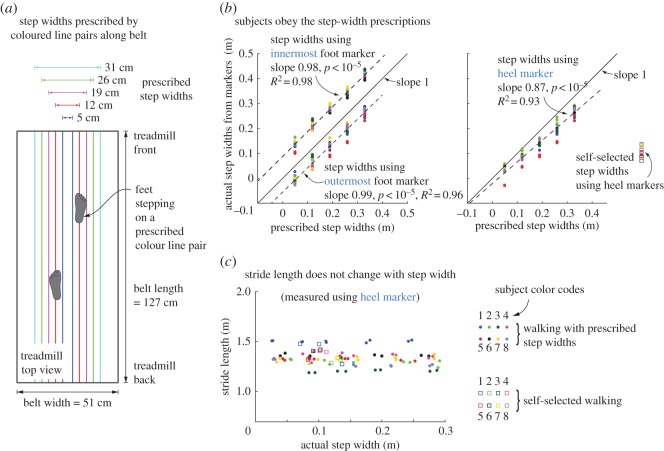
Experimental protocol. (*a*) Subjects walked at prescribed step widths by stepping on pairs of coloured lines drawn lengthwise along the treadmill belt. (*b*) Trial-wise averages of actual step widths are shown as a function of prescribed step widths. Actual step widths are computed three different ways: using the heel marker, using the innermost marker on each foot and using the outermost marker on each foot. Prescribed step widths are bracketed by the step widths computed using innermost and outermost markers. Subjects changed their step width linearly with prescribed step widths. Different colours indicate different subjects. Linear fit shown is to data pooled over all subjects. (*c*) Stride lengths were not significantly different for different prescribed step widths and when compared with normal walking. Different colours indicate different subjects, with the subject colour codes as shown.

Eight subjects participated in the study: five males and three females, with height 1.72±0.11 m, age 26.5±3.25 years and mass 70.3±9.85 kg (mean ± s.d.). Each subject walked on a treadmill at a constant belt speed of 1.25 ms^−1^ for 12 trials, each trial lasting 1–2 min. Two of these trials required normal walking—that is, with self-selected step widths. All other trials required walking at one of five prescribed step widths: 5, 12, 19, 26 and 33 cm, two trials per prescribed step width. Pairs of thin coloured lines drawn along the length of the treadmill prescribed these step widths (figure 1*a*). Subjects were instructed to walk placing their foot directly on top of the specified lines, with the lines falling roughly in the middle of their foot. The treadmill width of 0.51 m (figure 1*a*) is much larger than the largest prescribed step width. The trials had 210±50 steps (mean ± s.d.). Out of the 80 planned prescribed step-width trials and 16 planned self-selected walking trials across the eight subjects, we were able to use 75 prescribed step-width trials and 13 self-selected walking trials. The rest had corrupted data or their duration was too short for analysis.

Walking motion was recorded using a marker-based motion capture system (Vicon T20, 100 Hz, position error less than 0.3 mm). Subjects had four markers representing the torso: one anterior (roughly at the sacral level) and three posterior (whose average vertical position was roughly at the sacral level). They had four markers on each foot: heel, toe and two more on the lateral side of the foot roughly equally spaced between heel and toe. During analysis, the three-dimensional motion of the body, represented by the 12 markers, was simplified down to three points, one each for the torso, right foot and left foot (as in [6]). Each foot was represented simply by its heel marker, unless otherwise specified. The torso point was an average of the four torso markers to approximate the subjects' centre of mass. The marker-averaging had equal weights in the vertical and sideways direction, but in the fore–aft direction, the three posterior markers each had one-third the weight of the anterior marker.

### 2.2. Foot placement linear model

As in [6], we fit a linear model between the torso state at mid-stance (input) and the next foot position (output). In the following, *X* is the sideways direction (with rightwards positive), *Y* is the fore–aft or anterior–posterior direction (with forward positive) and *Z* is the vertical direction (with upward positive). Mid-stance is defined for each stride when the *Y* position on the torso is equal to that of the stance foot. We refer to a mid-stance as a ‘left mid-stance’ if the left foot is in stance during the mid-stance. We define ‘right mid-stance’ analogously. For each step, the origin of the coordinate system is the current stance foot position during mid-stance. This origin moves with the treadmill belt. We measure the torso state and the next foot position with respect to this coordinate frame. The torso state at mid-stance *T* is assumed to consist of the sideways position, sideways velocity and forward velocity, that is, $T=(X_{\mathrm{torso}},{\dot{X}}_{\mathrm{torso}},{\dot{Y}}_{\mathrm{torso}})$. The next stance foot position is *Q*=(*X*_foot_,*Y*_foot_). We seek a linear model of the form

2.1 $$Q-Q^{*}=J\;(T-T^{*})\;\mathrm{or}\;\Delta Q=J\;\Delta T,$$

where Δ*T* and Δ*Q* are the deviations of variables *T* and *Q* from their mean values T* and Q* over the whole trial and *J* is the 2×3 Jacobian matrix of sensitivities (partial derivatives) of the next stance foot position with respect to the torso state. The matrix *J* is estimated for each trial using ordinary least-squares regression. We ignored the vertical state variables *Z*_*torso*_ and ${\dot{Z}}_{\mathrm{torso}}$ in input *T* because they contribute less to explaining foot placement variability [6]. We ignored the fore–aft position *Y*_torso_ because it is zero at mid-stance, by definition. In addition to computing this linear model between *T* and *Q*, we also compute standard deviations of these quantities over each trial to quantify ‘kinematic variability’.

The individual coefficients in the linear model are denoted by partial derivative notation. For instance, $\partial X_{\mathrm{foot}}/\partial {\dot{X}}_{\mathrm{torso}}$ denotes the sensitivity of foot placement (*X*_foot_) to sideways deviations in sideways torso marker velocity (${\dot{X}}_{\mathrm{torso}}$) at mid-stance.

### 2.3. Poincare map and Floquet multipliers

Analogous to the linear model for the foot placement, we compute the linear model relating the torso state *T*_*i*_ at the *i*th left mid-stance and the torso state at the (*i*+1)th left mid-stance:

2.2 $$(T_{i}-T^{*})=P_{\mathrm{left}\to \mathrm{left}}\cdot (T_{i+1}-T^{*}).$$

We also computed the analogous mapping from the torso state from one right mid-stance to the next $P_{\mathrm{right}\to \mathrm{right}}$. These linear models are linearizations of the so-called Poincare map, also called the stride map or the first return map. The Poincare map describes how small deviations from the mean state get transformed over one stride; that is, one period of the gait cycle [18,16]. To compute these Poincare maps, we first computed the individual mappings for each of the two steps; that is, a mapping $P_{\mathrm{left}\to \mathrm{right}}$ from the left mid-stance state to right mid-stance state and a mapping $P_{\mathrm{right}\to \mathrm{left}}$ from the right mid-stance state to left mid-stance state. We then computed the full Poincare map by multiplying these individual step maps in appropriate order to obtain $P_{\mathrm{left}\to \mathrm{left}}$ or $P_{\mathrm{right}\to \mathrm{right}}$. Here, we use the same three-variable torso state $T=(X_{\mathrm{torso}},{\dot{X}}_{\mathrm{torso}},{\dot{Y}}_{\mathrm{torso}})$ as for the foot placement linear model. Thus, *P*s are 3×3 matrices. The three eigenvalues of the matrix *P* are called Floquet multipliers, which quantify what fraction of small state deviations remains after one stride. Smaller absolute values of these eigenvalues (Floquet multipliers) imply faster recovery from perturbations. Stable walking is implied by all eigenvalues having absolute values less than one.

### 2.4. Multiple comparisons using bootstrap statistics

We test whether the following 16 quantities show a linear trend (whether increasing or decreasing) with increasing step width:

— six foot placements control gains (elements of the 2×3 matrix *J* in equation (2.1)),
— two standard deviations of foot placement (one each for *X*_foot_ and *Y*_foot_),
— three standard deviations of mid-stance torso state (one each for *X*_torso_, ${\dot{X}}_{\mathrm{torso}}$, and ${\dot{Y}}_{\mathrm{torso}}$),
— two *R*^2^ values, corresponding to fractions of foot placement variance explained by the linear model in equation (2.1) (one each for predicting *X*_foot_ and *Y*_foot_) and
— three Floquet multipliers (eigenvalues of the 3×3 matrix *P* in equation (2.2)).

**Figure 2.**
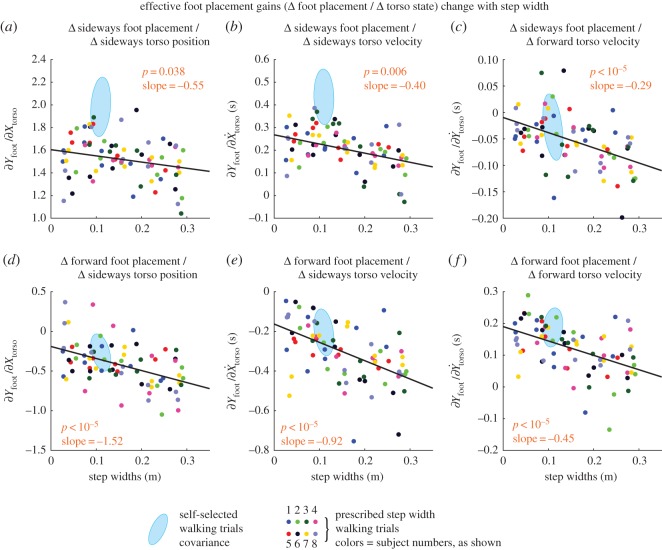
Foot placement control gains change with step width. All six foot placement control gains have a linear trend, all but one linear trend having *p*<0.0006 (*p*-values shown in figure). The foot placement control gains that couple sideways torso state to sideways foot placement, or fore–aft torso states to fore–aft foot placement, show a decreasing trend. The foot placement control gains that couple sideways and fore–aft directions show an increase in magnitude. The slope and the *p*-values are for the linear fit to step width. Low *p*-values suggest that there is a linear dependence between the each quantity and step width. The light blue ellipse shows the corresponding gains for self-selected walking and denotes the 1 s.d. covariance ellipse. Quantities with no units displayed are non-dimensional. Step widths shown are measured rather than prescribed. The foot placement gains shown here relate the left mid-stance torso deviations to the next (right) foot placement. Electronic supplementary material, figure S1 shows the contralateral analogue of this figure.

**Figure 3.**
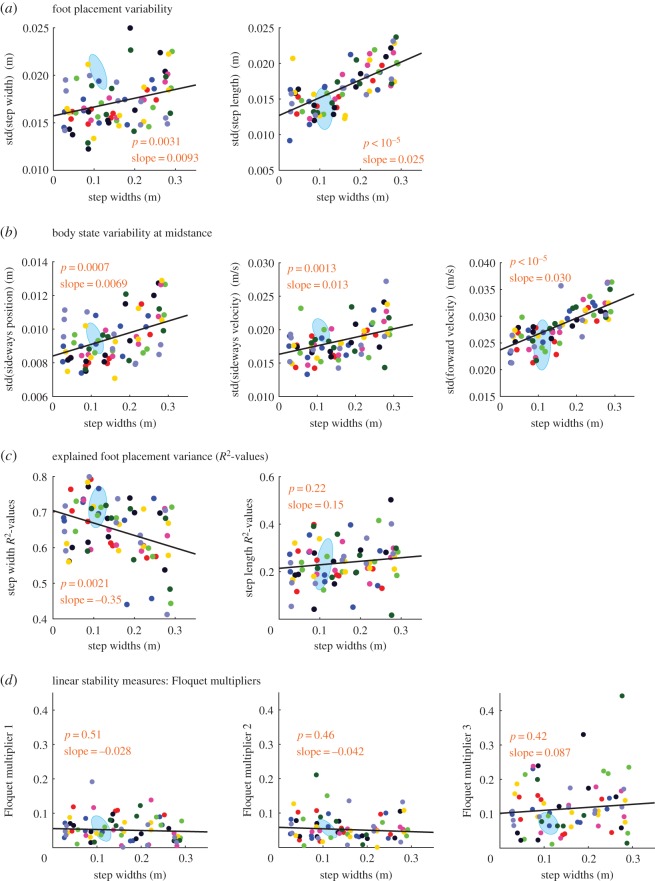
Torso state variability, foot placement variability, explained foot placement variance, and linear stability. (*a*) Foot placement variability (standard deviation) at the right foot relative to the previous left foot. Step-width variability is lower when step width is prescribed than in self-selected walking. Step length variability is higher when step width is prescribed than in self-selected walking. Both step width and step length variability increase with prescribed step width. (*b*) The standard deviations (variability) of torso sideways position and velocity at left mid-stance increases with increasing step width. (*c*) Fraction of right foot-placement variance explained by the linear model from mid-stance torso state (*R*^2^-values). (*d*) The Floquet multipliers for a three state variable map from one left mid-stance to the next left mid-stance do not change significantly much with respect to step width, nor are they significantly different from those during self-selected walking. The *p*-values and slopes shown in the figure panels are for the fit linear trends. Low *p*-value suggest a non-trivial linear trend. The light blue ellipse shows the corresponding quantities for self-selected walking and denotes the 1 s.d. covariance ellipse. Step widths shown are measured rather than prescribed. Quantities with no units displayed are non-dimensional. This figure corresponds to the states and mappings at left mid-stances (panel *b*), the following right foot placements (panels *a* and *c*), and mappings from right mid-stance to itself (panel *d*). Electronic supplementary material, figure S2 shows the contralateral analogue of this figure.

We use bootstrap statistics to compute the *p*-values for linear trends (non-zero slopes) in these 16 quantities, adjusted automatically for multiple comparisons [19–21]. This bootstrap procedure accounts for correlations between the various variables, as opposed to a simple Bonferroni correction, which is overly conservative [20]. Briefly, the bootstrap procedure is: (i) compute the 16 quantities for each of the walking trials; (ii) determine the best-fit linear relation between each quantity and prescribed step width; (iii) subtract out the linear trend to compute the residuals for each of the 16 quantities, giving us data satisfying the null hypothesis (that is, no linear trends [21]); (iv) construct 10^5^ bootstrap samples by re-sampling from this data with no linear trends; this re-sampling preserves correlations between the 16 quantities; (v) for each bootstrap sample, obtain the best linear fits for each of the 16 quantities; (vi) the *p*-value for each of the 16 slopes is estimated as the fraction of bootstrap samples for which the linear trend's slope is as great as that found with the original sample [20,21]. Say, λ is the slope from the original data (before linear trend subtraction) and $\bar{\lambda }$ is the slope obtained from the data without a linear trend. Then, this *p*-value is the probability that $\bar{\lambda }$ is at least as great as λ, just by random chance, even though there is no linear trend. When none of the 10^5^ bootstrap samples exhibit a slope as large as in the original sample, *p*<10^−5^ is the best estimate of the *p*-value we can provide. So, in this case, we simply state that *p*<10^−5^. When computing the best-fit linear models of these quantities with respect to step width, we allow for different constant terms (vertical offsets) for each subject, but the same slope for all subjects. In figures 2 and 3 and electronic supplementary material, figures S1–S2, we show the linear trend corresponding to the mean constant term over all subjects; the scatter plots for each subject are translated by the amount by which the mean offset differs from the subject-specific offset.

## 3. Results

The subjects obeyed the step-width constraint, in that the step widths computed using heel markers were linearly related to the prescribed step width (figure 1*b*). Further, for every trial, the prescribed step width was always between step width computed using the outermost markers (most lateral) and the step width computed using the innermost marker (most medial). This suggests that subjects did step on the coloured lines as instructed (figure 1*b*).

When step width was prescribed, the mean stride lengths did not change significantly as a function of step width (a linear model on step widths had *p*=0.17 compared to a constant model). Furthermore, these stride lengths when step width was constrained were not significantly different from stride lengths during self-selected walking (unpaired *t*-test, *p*=0.14, figure 1*c*).

All foot placement control gains (coefficients in the linear model for foot placement) that map torso deviations to foot placement change systematically with changing prescribed step width, (see figure 2 and electronic supplementary material, figure S1). More specifically, the foot placement gains that couple sideways to sideways (figure 2*a*,*b* and electronic supplementary material, figure S1a,b) or fore–aft to fore–aft (figure 2*f* and electronic supplementary material, figure S1f) all systematically decrease. The foot placement gains that couple sideways and fore–aft (figure 2*c*,*d*,*e* and electronic supplementary material, figure S1c,d,e) all increase in magnitude.

Sideways foot placement control gains corresponding to sideways torso deviations are much lower when step width is prescribed versus when step width is self-selected (figure 2*a* and electronic supplementary material, figure S1a). Thus, it appears that as soon as step width is prescribed, subjects use step-width modulation much less than during normal walking. Similarly, as might be expected, when step-width was prescribed, the step-width variability was lower than during normal self-selected walking (*p*=0.005, figure 3*a* and electronic supplementary material, figure S2a).

The linear model for foot placement as a function of torso state explained about 70% of the sideways foot placement variance and 25% of the fore–aft foot placement variance over all conditions (figure 3*c* and electronic supplementary material, figure S2c). The fraction of step-width variance explained decreased with increasing step-width. Furthermore, the fraction of the step-width variance explained at the highest step widths was lower than during normal self-selected walking. This suggests that the subjects were using foot placement less in response to torso state deviations.

The step-width variability increased slightly with increasing step width, but step length variability increased much more consistently with increasing prescribed step width (figure 3*a* and electronic supplementary material, figure S2a). The variability in torso state $\left(X_{\mathrm{torso}},{\dot{X}}_{\mathrm{torso}},Y_{\mathrm{torso}}\right)$ also showed a linear trend with step width (figure 3*b* and electronic supplementary material, figure S2b, *p*<0.05). Furthermore, we found that our simple measure of linear stability, namely the Floquet multipliers either did not appreciably change with increased step width or had a very weak increasing trend (figure 3*d* and electronic supplementary material, figure S2d). Thus, it appears that walking with increased step width does not increase how quickly perturbations decay. It seems as though humans maintain roughly the same level of linear stability independent of the prescribed step width. This result is contrary to the *a priori* expectation about step width increasing stability.

## 4. Discussion

The idea that wider stance confers some stability advantages has some precedence [22,23]. For instance, having a wide stance may increase stability especially in standing [24]. Analogously, in the case of some animals, a sprawled posture [15,25] increases stability. ‘Cautious walking’ is also sometimes described as having larger step widths and slower speeds [12]. Elderly and those prone to falling walk with larger step widths, perhaps to compensate for other factors that decrease their walking stability [22,26]. (They also often have increased step-width variability [27].) Of course, this possibly increased stability from having a wide base of support may come at the cost of higher energy cost [28].

Here, we have shown that the foot placement control gains change systematically with increased prescribed step widths. That is, when the prescribed step width is higher, humans modulate their foot placement less (both fore–aft and sideways) in response to a given torso state deviation in the same direction. We speculate that this reduced foot placement modulation may be because the wider mean foot placement may already confer some stability, so that humans need to adjust their foot placement less. For instance, if a person walking with a large step width experiences a rightward perturbation, a corrective leftward force is provided by the already wide step and thus there is no need to widen it further. Of course, this heuristic reasoning does not explain the systematical changes in the other foot placement gains. Alternatively, the changes in foot placement modulation may be because of the systematic changes in the mean walking motion that accompanies the step-width increase.

The sideways foot placement gains in response to sideways torso deviations were generally lower than during normal walking, even controlling for step width. Thus, just prescribing the step width using a line drawn on the ground affects how much foot placement is used in the walking dynamics. Thus, some changes in foot placement modulation may be due to the foot placement being now under partly conscious control, mediated by visual feedback. An alternative hypothesis for reduced foot placement control could be that prescribing the step width serves simply as a distraction. This alternative hypothesis could be tested by using other distractors, for instance, increased cognitive load [29].

It may seem oxymoronic to examine step-width modulation when step-width is ‘prescribed’ via the lines on the ground. But our prescription of the step-width is not an inviolable constraint. Satisfying this step-width constraint still allows the subject considerable step-width variability as shown in figure 3*a*. Furthermore, the step-width variability has a systematic trend that cannot be explained just by the fact that the step width is prescribed. The subjects presumably deviate from the prescribed step width slightly to take advantage of usefulness of foot placement modulation in stable walking.

An alternative way to make subjects walk with a larger step width is to simply ask them to walk with a larger step width, without precisely specifying the desired step width. Under such an instruction in [12], the standard deviation of the subjects' step widths increased with step width, as in our study. With this alternative increased-step-width protocol [12], the step-width standard deviation was over 35 mm on average compared with only about 18 mm on average in our study. This much larger standard deviation is presumably because the step width was not precisely prescribed. Using this alternative protocol, some alternative measures related to stability were examined in [12,13], but not the foot placement control gains; similar trends were observed for variability changes.

Our results suggest that while increasing step width may change how we modulate foot placement, it does not much affect the simple linear stability measure we considered, namely, Floquet multipliers. It appears as though humans are expending less control effort in foot placement modulation to maintain the same level of overall stability. Unfortunately, stability is not quantified with a single quantity. The Floquet multipliers only tell us how quickly small deviations are corrected. They cannot directly tell us about the likelihood of falling during normal walking or due to a large external perturbation. Understanding such alternative aspects of stability would require better characterizing the endogenous noise during normal walking and determining the largest perturbation from which humans can recover. Further, we have estimated the dominant Floquet multipliers here by assuming a three state-variable model, but using a higher dimensional model may require walking experiments with a sufficiently rich set of perturbations.

The results presented here may be applicable to visual presentations of foot placement cues in rehabilitation scenarios, for instance, in the context of mitigating freeze-gait in Parkinson patients or improving balance in stroke patients, using simple projections on the floor or augmented reality visors [30–32]. Future work may involve inferring a more detailed controller from the step width controlled walking data, using more state variables in the analysis, characterizing how foot placement is modulated through a mixture of feedforward and feedback control [33], attempting to explain the observed trends using optimal feedback control, repeating the experiments with a wider band to step on (rather than a line) [31], repeating the experiments with an instruction to walk with wider or narrower step widths [12], and examining stability using perturbation experiments.

## Supplementary Material

Supplementary Information

## Data accessibility

Data and code are available at Dryad: http://dx.doi.org/doi:10.5061/dryad.1fh71 [34] and at M.S.'s website: http://movement.osu.edu/webpages_for_papers/StepWidthJennifer2016.html.
