# Correction to ‘Walking with wider steps changes foot placement control, increases kinematic variability and does not improve linear stability’

**DOI:** 10.1098/rsos.172000

**Published:** 2018-01-10

**Authors:** Jennifer A. Perry, Manoj Srinivasan

*R. Soc. open sci.* **4**, 160627. (Published Online 13 September 2017). (doi:10.1098/rsos.160627)

In figure 1*b*, the labels for the two dashed lines (and the corresponding data scatter) should be interchanged and modified as shown in the following revised figure 1. The upper dotted line should have the following label: ‘step widths using outermost foot marker. Slope 0.99, *p *< 10^−5^, *R*^2^ = 0.98’. The lower dashed line should have the following label: ‘step widths using the innermost foot marker. Slope 0.98, *p *< 10^−5^, *R*^2^ = 0.96’.

**Figure 1.**
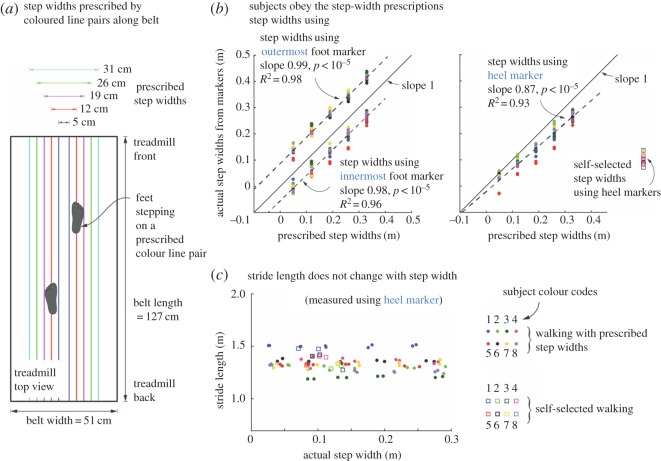
(*Overleaf*.) Experimental protocol. (*a*) Subjects walked at prescribed step widths by stepping on pairs of coloured lines drawn lengthwise along the treadmill belt. (*b*) Trial-wise averages of actual step widths are shown as a function of prescribed step widths. Actual step widths are computed three different ways: using the heel marker, using the innermost marker on each foot and using the outermost marker on each foot. Prescribed step widths are bracketed by the step widths computed using innermost and outer most markers. Subjects changed their stepwidth linearly with prescribed step widths. Different colours indicate different subjects. Linear fit shown is to data pooled over all subjects. (*c*) Stride lengths were not significantly different for different prescribed step widths and when compared with normal walking. Different colours indicate different subjects, with the subject colour codes as shown.

The *y*-axis labels in figure 2*a*–*c* were incorrect in the published paper. Furthermore, the *p-*value in panel (*b*) should be 0.0006. The *y*-axis for figure 2*d*–*f* were presented correctly. The correct figure 2 and an updated figure captions are shown below.

**Figure 2.**
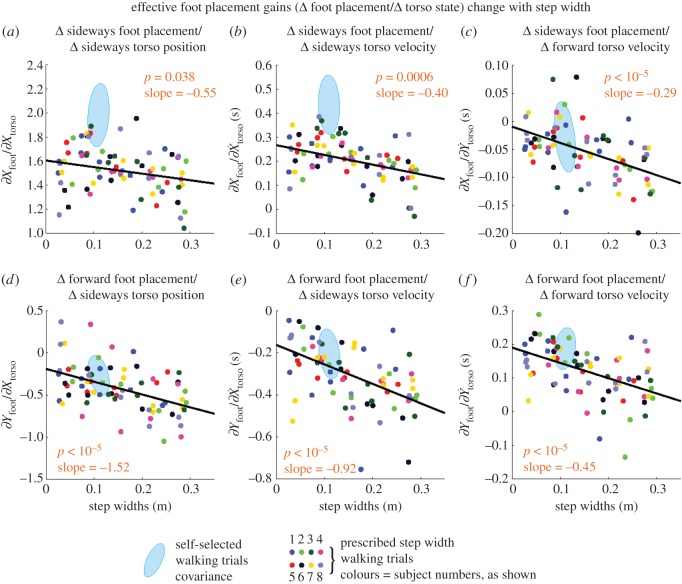
Foot placement control gains change with step width. All six foot placement control gains have a linear trend, all but one linear trend having *p* ≤ 0.0006 (*p*-values shown in figure). The foot placement control gains that couple sideways torso state to sideways foot placement, or fore–aft torso states to fore–aft foot placement, show a decreasing trend. The foot placement control gains that couple sideways and fore–aft directions show an increase in magnitude. Negative gains increase in magnitude by decreasing in value, with a negative slope. The slope and the *p*-values are for the linear fit to step width. Low *p-*values suggest that there is a linear dependence between the each quantity and step width. The light blue ellipse shows the corresponding gains for self-selected walking and denotes the 1 s.d. covariance ellipse. Quantities with no units displayed are non-dimensional. Step widths shown are measured rather than prescribed. The foot placement gains shown here relate the left mid-stance torso deviations to the next (right) foot placement. Electronic supplementary material, figure S1 shows the contralateral analogue of this figure.

These typographical errors do not affect any of the scientific conclusions of the article.

