# Supplementary material for: Walking with wider steps changes foot placement control, increases kinematic variability and does not improve linear stability
Source: R Soc Open Sci. 2017 Sep 13;4(9):160627. doi: 10.1098/rsos.160627 (PMC5627068; doi:10.1098/rsos.160627)
Supplement: Supplementary Information [file rsos160627supp1.pdf]

Supplementary Information for: Walking with wider steps changes foot placement control, increases kinematic variability, and does not improve linear stability

Jennifer A. Perry and Manoj Srinivasan
Mechanical and Aerospace Engineering, The Ohio State University

Figures 2 and 3 in the main manuscript provide details about torso state at a left mid-stance, right foot placement, and a stride map starting and ending at the right mid-stance. The two figures S1 and S2 in this supplementary information appendix provide the 'contralateral analog' of Figures 2-3 of the main manuscript.

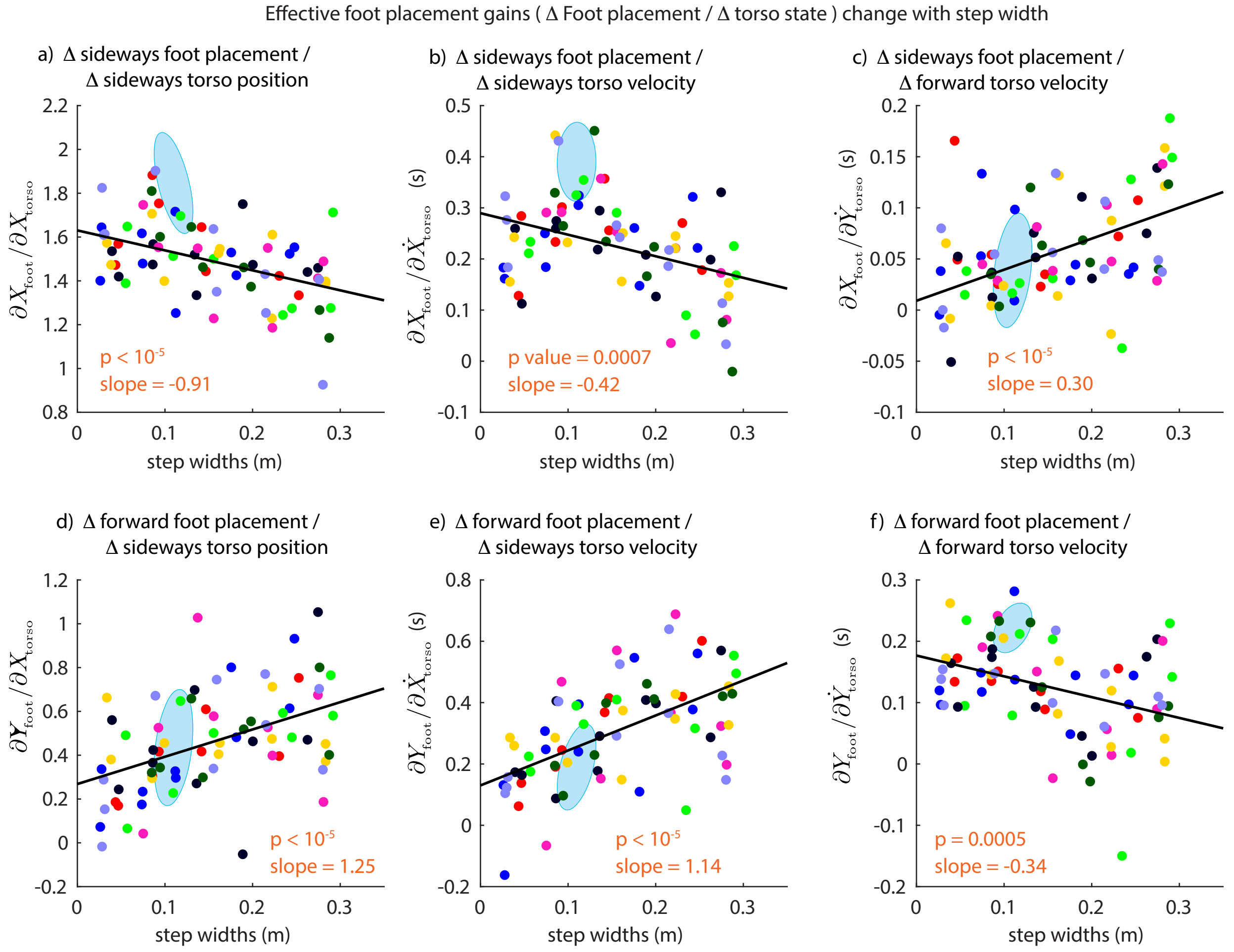

Figure S1: Effective foot placement gains for the left foot. This figure is the contralateral analog of Figure 2 of the main manuscript. It shows the feedback gains for how left foot placement is modulated in response to torso state deviations at the previous right mid-stance. The foot placement control gains that couple sideways torso states to sideways foot placement, or fore-aft torso state to fore-aft foot placement, show a decreasing trend. The foot placement control gains that couple sideways and fore-aft directions show an increase in magnitude. The p values and slopes correspond to the linear fit to the gains versus step width. Low p values suggest that there is a linear dependence between the each quantity and step width. Light blue ellipses shows the gains for self-selected walking and denotes the one standard deviation covariance ellipse. Quantities with no units displayed are non-dimensional. Step widths shown are measured rather than prescribed. Different colored dots indicate different subjects.

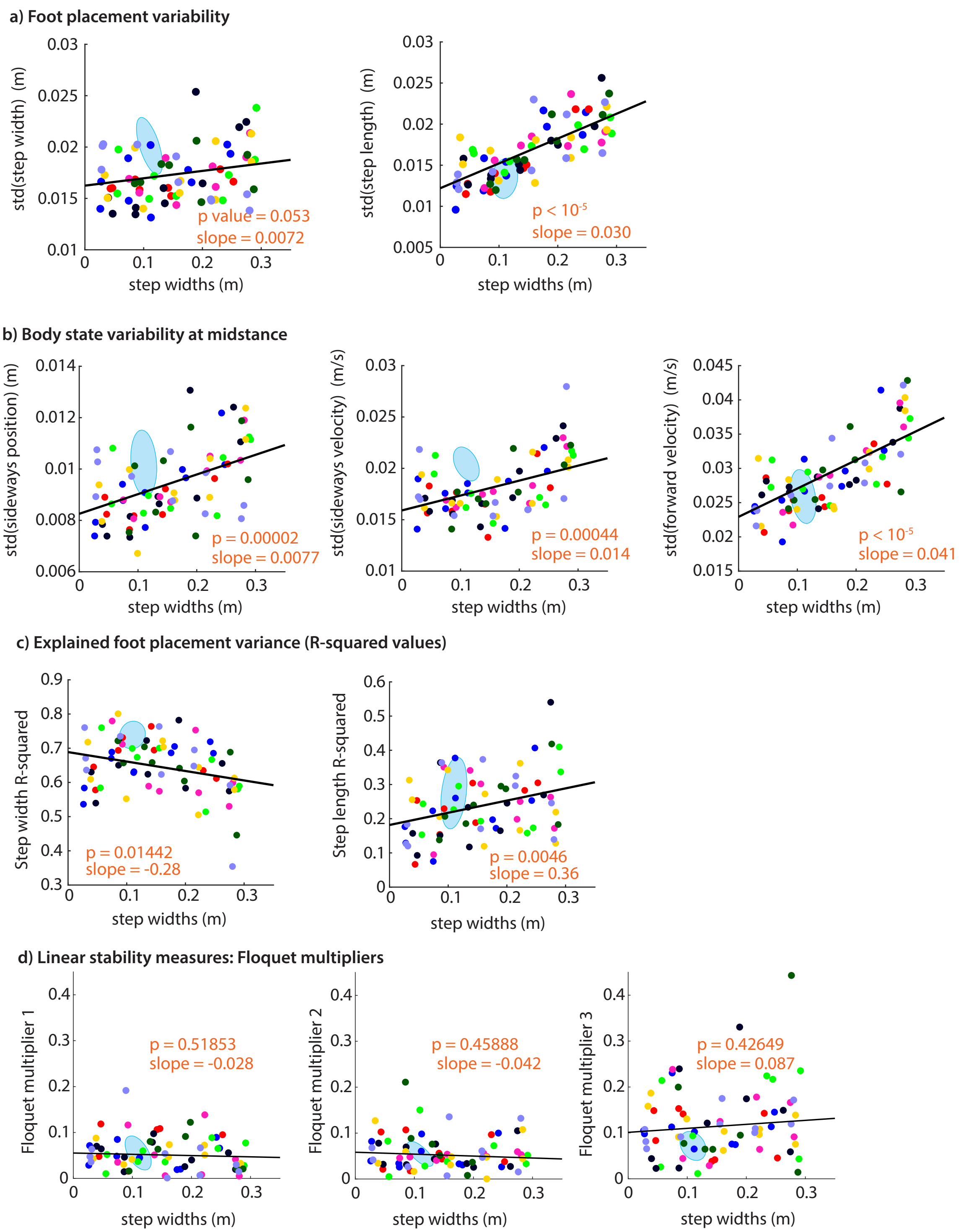

Figure S2: Torso state standard deviations, foot placement standard deviations, explained foot placement variance, and linear stability. This figure is the contralateral analog of Figure 3 of the main manuscript. a) Foot placement variability at the left foot relative to the previous right foot. b) Body state variability at the right mid-stance. c) Fraction of left foot placement variance explained by linear model based on previous mid-stance torso state. d) Floquet multipliers are computed from the torso state mapping from a left mid-stance to the next left mid-stance. Different colored dots indicate different subjects. The p values and slopes correspond to the linear fit to the gains versus step width. Low p values suggest that there is a linear dependence between the each quantity and step width. Light blue ellipses shows the gains for self-selected walking and denotes the one standard deviation covariance ellipse.
